# Severe Early-Onset Pulmonary Hypertension in a Six-Month-Old With Down Syndrome and Isolated Secundum Atrial Septal Defect

**DOI:** 10.7759/cureus.84019

**Published:** 2025-05-13

**Authors:** Fatima Abeer, Aasim Ayaz Wani, Bisma Javid, Aisha Mahmood, Gazala Andleeb

**Affiliations:** 1 Department of Internal Medicine, Government Medical College, Srinagar, Srinagar, IND; 2 Department of Engineering, National Institute of Technology Srinagar, Srinagar, IND; 3 Department of Pediatrics, Government Medical College, Srinagar, Srinagar, IND

**Keywords:** atrial septal defect, congenital heart disease, down's syndrome, early-onset pulmonary hypertension, pediatric pulmonary hypertension, pulmonary arterial hypertension in infants

## Abstract

Infants with Down syndrome (trisomy 21) commonly present with congenital heart defects and immune dysregulation, significantly increasing the risk of early-onset pulmonary arterial hypertension (PAH). Although secundum atrial septal defects (ASDs) are often considered hemodynamically mild in non-syndromic children, they can progress aggressively in the presence of trisomy 21. We describe a six-month-old male infant with karyotype-confirmed trisomy 21 who developed severe PAH secondary to a rapidly enlarging secundum ASD - a highly atypical presentation for an isolated lesion. The infant presented with fever, respiratory distress, vomiting, and diarrhea, alongside a clinical history of neonatal sepsis, recurrent infections, failure to thrive (weight below the 5th percentile), and subclinical hypothyroidism (TSH 8.12 μIU/mL). Echocardiography revealed that the ASD had enlarged from 6 mm at five months to 10 mm, creating a substantial left-to-right shunt (Qp:Qs >1.5:1). Management with IV ceftriaxone, sildenafil (2 mg twice daily), supplemental oxygen, and nutritional support stabilized the infant within five days (SpO₂ 93-94% on room air). He was discharged for deferred surgical ASD closure, highlighting the value of early pulmonary vasodilator therapy as a bridge to definitive repair. This case underscores the markedly increased susceptibility of infants with Down syndrome to severe PAH, even in the setting of a seemingly hemodynamically insignificant ASD. Early cardiac evaluation, prompt intervention, and multidisciplinary management are crucial to preventing irreversible pulmonary vascular disease in this high-risk population.

## Introduction

Down syndrome (trisomy 21), which occurs in approximately 1 in 700 live births, is well recognized for its strong association with congenital heart defects (CHDs), notably atrial septal defects (ASDs), present in up to half of affected individuals [1]. In typical pediatric populations, secundum ASDs rarely cause significant clinical issues in early infancy. However, Down syndrome-related genetic and immunologic factors place these infants at disproportionately high risk for early-onset pulmonary arterial hypertension (PAH), often within the first year of life [2,3]. This susceptibility is driven by multiple trisomy 21-specific mechanisms, including overexpression of genes such as DYRK1A and RCAN1 (contributing to endothelial dysfunction), alveolar hypoplasia, and an exaggerated hemodynamic response to left-to-right shunts [4-6]. Accordingly, Down syndrome confers a nearly ten-fold increase in the likelihood of developing PAH compared to non-syndromic infants, with early-onset disease carrying a poor prognosis if not identified and treated promptly [4,7].

We describe a rare presentation of severe PAH in a six-month-old infant with Down syndrome and an isolated secundum ASD that expanded from 6 mm to 10 mm over the course of a single month. This rapid progression was accompanied by recurrent infections, including neonatal sepsis, and failure to thrive, culminating in life-threatening respiratory compromise. To our knowledge, this represents one of the earliest documented instances of severe PAH arising from an ostensibly uncomplicated ASD in an infant with Down syndrome, demonstrating the need for vigilant echocardiographic surveillance and timely intervention. We also discuss the potential role of pulmonary vasodilators, specifically sildenafil, as a bridge to surgical closure in medically fragile infants, emphasizing the delicate balance between the benefits of early repair and the risks associated with operating on clinically unstable patients.

## Case presentation

Background and neonatal history

**Figure 1:**
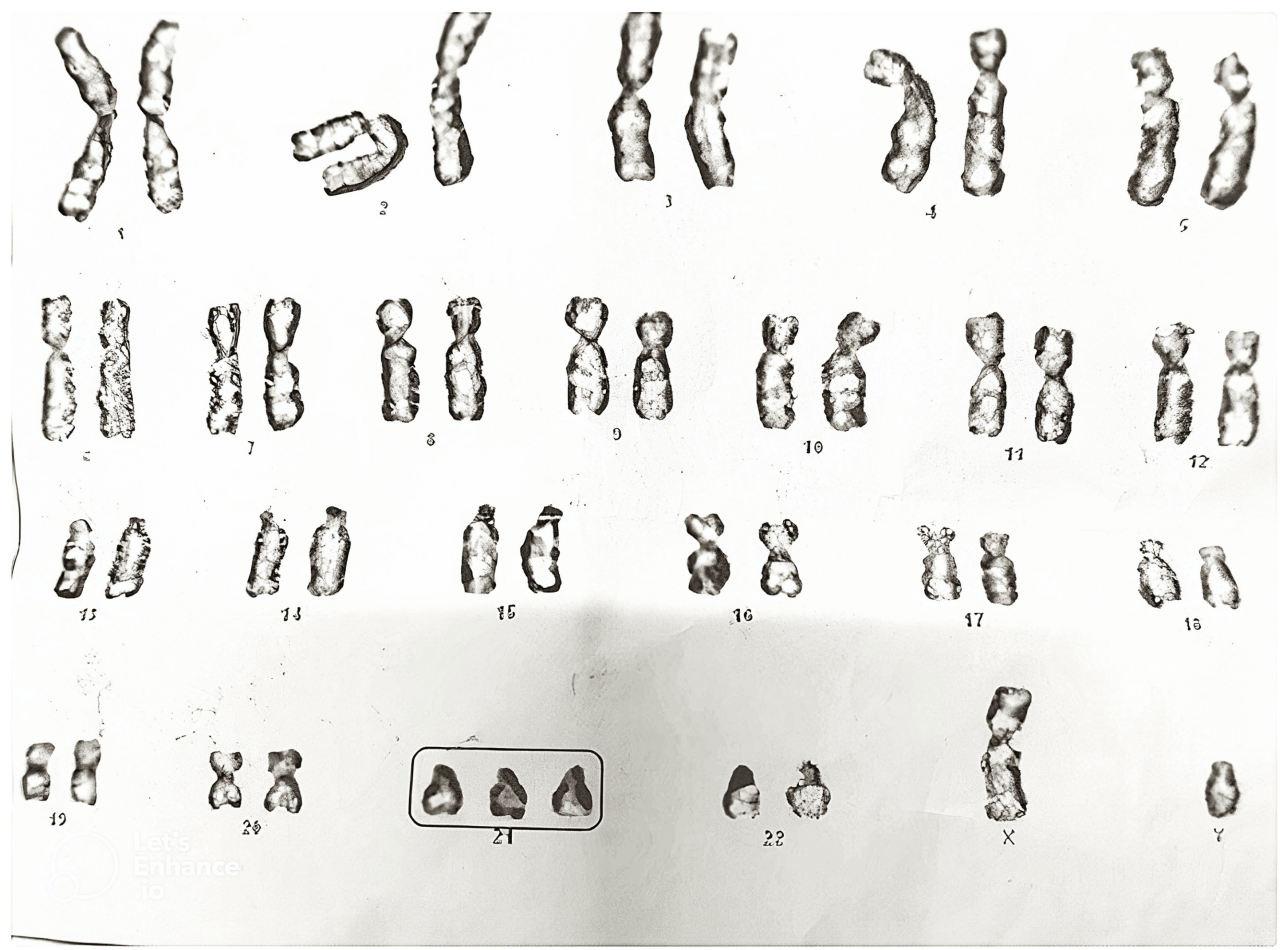
The image displays a complete chromosomal complement arranged in pairs from chromosomes 1 to 22, along with sex chromosomes (XY). Notably, there are three copies of chromosome 21 (highlighted in the box), consistent with Trisomy 21 (Down syndrome). This chromosomal abnormality is the most common genetic cause of intellectual disability and is frequently associated with congenital heart defects, hypotonia, characteristic facial features, and increased risk of pulmonary hypertension.

A six-month-old male infant, born full-term by cesarean section to non-consanguineous parents, displayed characteristic phenotypic features of Down syndrome (DS) at birth, including hypotonia, upslanting palpebral fissures, a flat facial profile with epicanthic folds, a protruding tongue, and a broad neck. Although karyotyping was pending early on, the clinical diagnosis of trisomy 21 was evident (see Figure 1). His birth weight was 2.8 kg (slightly below average).

Growth and early morbidities

From the neonatal period, the infant exhibited poor weight gain, weighing only 3.5 kg at six months (<5th percentile for age) and often tiring during feeds. At 17 days of life, he developed omphalitis that progressed to neonatal sepsis, necessitating IV antibiotics and supportive care; no cardiovascular assessments (such as an echocardiogram) were performed at that time. Recurrent mild respiratory infections occurred throughout infancy, though no formal pneumonia diagnosis was made until the current illness.

Presenting illness

In early March 2025, the infant arrived at the pediatric emergency department with one day of high-grade fever (up to 39°C), frequent vomiting, and multiple episodes of watery diarrhea. The mother reported increased work of breathing and poor feeding, with the infant appearing irritable, lethargic, and far less active than usual. There were no recent travel exposures or known tuberculosis contacts. Immunizations were age-appropriate.

Physical examination

On arrival, the infant was febrile (38.5°C), tachypneic (respiratory rate 80-85/min), and tachycardic (heart rate 150/min), with room-air oxygen saturation of 88%. Both his weight (3.5 kg) and length (60 cm) fell below the 5th percentile, confirming failure to thrive. Dysmorphic features consistent with Down syndrome were evident (e.g., flat occiput, single transverse palmar crease), and generalized hypotonia was noted. Respiratory findings included subcostal retractions, nasal flaring, and bibasilar crackles, indicating pneumonia. Cardiovascular examination revealed a hyperdynamic precordium and a right ventricular heave, with a grade 3/6 systolic ejection murmur at the left upper sternal border. The murmur was accompanied by wide, fixed splitting of the second heart sound (S2) - a hallmark of atrial septal defect (ASD) physiology. A loud pulmonic component (P2) of S2 suggested pulmonary hypertension (PH). Mild hepatomegaly (liver 3 cm below the right costal margin) was also detected, likely reflecting right-sided volume overload.

Initial investigations

(a) Routine laboratory tests indicated leukopenia (WBC 4.2×10^9^/L) with a slightly low absolute neutrophil count (~1.5×10^9^/L), moderate anemia (Hb 9.0 g/dL), and a markedly elevated C-reactive protein (CRP) (234 mg/L), suggestive of significant infection or sepsis. Liver enzymes were mildly elevated [Aspartate transaminase (AST) 57 U/L, alanine transaminase (ALT) 105 U/L] without hyperbilirubinemia, compatible with either reactive hepatitis or mild congestive hepatopathy (see Table 1). Blood and urine cultures were negative, presumably due to prompt antibiotic administration. A stool sample was negative for common bacterial pathogens. (b) Thyroid function tests showed a thyroid-stimulating hormone (TSH) of 8.12 μIU/mL with normal free T4 - diagnostic of subclinical hypothyroidism (a recognized comorbidity in Down syndrome). Given the infant’s acute condition and normal T4, endocrine follow-up was planned rather than immediate hormone replacement. (c) Chest radiography demonstrated bilateral perihilar infiltrates (consistent with bronchopneumonia), along with cardiomegaly and a prominent pulmonary artery segment - raising suspicion of a significant cardiac shunt and pulmonary hypertension (see Table 2).

**Table 1:** Complete Blood Count and Differential (27/07/2024) MCV: Mean Corpuscular Volume; MCH: Mean Corpuscular Hemoglobin; MCHC: Mean Corpuscular Hemoglobin Concentration; RDW-CV: Red Cell Distribution Width - Coefficient of Variation; MPV: Mean Platelet Volume

Parameter	Value	Unit	Reference Range	Interpretation
WBC	5.18	×10³/μL	5.0 – 19.5	Low-normal
Neutrophils	34.1	%	20 – 40	Normal
Lymphocytes	49.4	%	40 – 70	Normal
Monocytes	9.7	%	2 – 10	High-normal
Eosinophils	6.1	%	1 – 4	↑ Mild eosinophilia
Basophils	0.7	%	<1	Normal
RBC	3.79	×10⁶/μL	4.1 – 5.5	↓ Anemia
Hemoglobin	9.7	g/dL	10.5 – 14.0	↓ Mild anemia
Hematocrit	28.6	%	32 – 44	↓ Low
MCV	75.6	fL	70 – 86	Normal
MCH	25.6	pg	23 – 31	Normal
MCHC	33.9	g/dL	30 – 35	Normal
RDW-CV	19.6	%	<14.5	↑ Anisocytosis
Platelet Count	438	×10³/μL	150 – 400	↑ Mild thrombocytosis
MPV	9.1	fL	6.5 – 10.5	Normal

**Table 2:** Basic Metabolic Panel and Arterial Blood Gas Parameters at Admission BUN: Blood urea nitrogen

Parameter	Value	Unit	Reference Range	Interpretation
pH	7.41	Unitless	7.35 – 7.45	Normal
pCO₂	40	mmHg	35 – 45	Normal
pO₂	43	mmHg	60 – 100	↓ Mild hypoxemia
Bicarbonate	18.0	mmol/L	22 – 28	↓ Metabolic acidosis
BUN	3.86	mg/dL	5 – 18	↓ Slightly low
Creatinine	0.39	mg/dL	0.3 – 0.7	Normal
Sodium	143	mmol/L	135 – 145	Normal
Potassium	4.6	mmol/L	3.5 – 5.5	Normal
Calcium	9.5	mg/dL	8.5 – 10.5	Normal
Phosphate	5.0	mg/dL	4.5 – 6.7	Normal
Bilirubin (T)	0.5	mg/dL	<1.2	Normal

Cardiac evaluation

An urgent transthoracic echocardiogram revealed a 10 mm secundum ASD with a left-to-right shunt (Qp:Qs >1.5:1), along with right atrial and ventricular enlargement. There was moderate tricuspid regurgitation, and Doppler estimates of right ventricular systolic pressure indicated severe pulmonary arterial hypertension (PAH). Retrospectively, an ASD measurement of 6 mm had been documented during a screening echocardiogram performed one month earlier as part of the routine cardiac evaluation recommended for infants with Down syndrome - already accompanied by elevated pulmonary pressures - suggesting rapid progression of pulmonary vascular pathology. No other structural cardiac anomalies were identified (e.g., no ventricular septal defect or patent ductus arteriosus).

Although the two echocardiograms were performed by different pediatric cardiologists, both assessments were conducted at the same center using standardized pediatric protocols. Minor inter-observer variability in measuring ASD size is possible, but the accompanying clinical deterioration, right heart dilation, and increased shunt volume suggest genuine progression in both anatomical and hemodynamic terms. While idiopathic PAH remains a differential consideration - especially in Down syndrome - the presence of a significant left-to-right shunt, radiologic signs of volume overload, and modest improvement in PA pressures following infection control collectively support ASD-related PAH as the primary etiology in this case. Although right heart catheterization and advanced workup (e.g., autoimmune or hematologic evaluation) were not performed due to the patient's clinical instability, no clinical or laboratory features suggested an alternative cause.

Working diagnosis

Combining clinical and investigational findings, the team arrived at the following key diagnoses: (a) Down syndrome (Trisomy 21) - with associated features and subclinical hypothyroidism (see Table 3). (b) Congenital heart disease - an isolated secundum ASD (~10 mm), now significantly shunting left-to-right. (c) Severe pulmonary arterial hypertension - presumably driven by the large ASD in the context of Down syndrome’s predisposition to early-onset PH. (d) Acute pneumonia and sepsis - evidenced by fever, respiratory distress, high CRP, and improvement on antibiotics. (e) Acute gastroenteritis - manifesting with vomiting and diarrhea, likely viral or antibiotic-related. (f) Failure to thrive - multifactorial (cardiac burden, recurrent infections, DS-related feeding challenges). (g) Subclinical hypothyroidism - common in DS, potentially contributing to poor growth and delayed metabolic adaptation.

**Table 3:** Thyroid profile obtained on 21/01/2025. The test revealed elevated thyroid-stimulating hormone (TSH) levels with normal T3 and T4, suggestive of subclinical hypothyroidism—a common endocrinological finding in infants with Down syndrome.

Parameter	Value	Unit	Reference Range	Interpretation
Total T3	1.78	ng/mL	1.05 – 2.45	Normal
Total T4	11.78	μg/dL	5.92 – 13.06	Normal
TSH	8.12	μIU/mL	0.70 – 6.40	↑ Subclinical hypothyroidism

Hospital course and management overview

Upon admission to the pediatric intensive care unit, antibiotic therapy (IV ceftriaxone) and supportive measures (oxygen supplementation, IV fluids for dehydration, careful monitoring of fluid balance) were initiated. Given severe PAH, sildenafil (a phosphodiesterase-5 inhibitor) was started at a low dose to reduce pulmonary artery pressure. The infant tolerated the regimen without hypotension and showed gradual clinical improvement by the third hospital day. Oxygen saturation increased from 88% to 93-94% on room air; however, this improvement likely reflected resolution of pneumonia rather than a direct effect of sildenafil, given the left-to-right nature of the shunt. Bronchodilators and antiemetics were administered as indicated, and nutritional support was optimized to address failure to thrive. By Day 5, fever resolution, improved WBC count (8×10^9^/L), and reduced CRP (50 mg/L) reflected infection control. A repeat echocardiogram continued to show severe PAH, though with a modest decrease in estimated pulmonary pressures. Definitive ASD closure was deemed essential to prevent progression to irreversible pulmonary vascular disease; however, the cardiac surgery team recommended delaying repair until the infant was more stable, had gained weight, and the risk of perioperative complications was lower.

## Discussion

This case illustrates the unusually rapid progression of pulmonary arterial hypertension (PAH) in a six-month-old infant with Down syndrome (DS) and an isolated secundum atrial septal defect (ASD) - a combination not typically associated with severe early-onset PAH. While ASDs are generally well tolerated in non-syndromic infants, DS confers a nearly 10-fold increased risk of PAH due to trisomy 21-specific genetic, immunologic, and pulmonary vulnerabilities [1,4]. Although rarely reported, similar cases in the literature describe early PAH in DS infants with pre-tricuspid shunts, suggesting that even isolated ASDs can be clinically significant in this population. Our patient represents one of the youngest documented cases of such progression, emphasizing the need for vigilant, high-frequency echocardiographic surveillance and multidisciplinary care to prevent irreversible pulmonary vascular disease.

Pathophysiology: a multifaceted cascade

Down syndrome confers unique genetic and anatomical vulnerabilities that intensify the hemodynamic impact of intracardiac shunts. Overexpression of genes such as DYRK1A and RCAN1 in trisomy 21 may promote smooth muscle proliferation and impair nitric oxide-mediated vasodilation, priming the pulmonary vasculature for hyper-reactivity [5,6]. Concurrently, alveolar simplification, impaired lymphatic drainage, and reduced pulmonary vascular capacity further predispose affected infants to earlier and more severe PAH [5]. In our patient, an ASD that expanded from 6 mm to 10 mm over just 1.5 months imposed a substantial left-to-right shunt on an already compromised pulmonary circuit. While inter-operator variability in echocardiographic measurements cannot be excluded, the consistent progression in both imaging and clinical status supports a true increase in shunt size and hemodynamic burden.

Environmental and clinical stressors further magnified this risk [7]. Additional factors, such as the concurrent pneumonia, likely worsened respiratory compromise and further elevated pulmonary pressures. However, it is important to recognize that diagnosing severe PAH during active respiratory infection is inherently challenging, as transient hypoxia and inflammation can acutely increase pulmonary artery pressures. In our case, the modest reduction in PA pressures following antibiotic therapy and pulmonary vasodilator initiation suggests that the infection acted as an acute exacerbating factor on top of an evolving pulmonary vascular pathology. Therefore, reassessment of PAH after clinical stabilization is critical to avoid overestimation and to determine the optimal timing for intervention.

The infant’s history of neonatal sepsis at 17 days of life likely triggered an early systemic inflammatory response, contributing to pulmonary vascular remodeling through hypoxic vasoconstriction and pro-inflammatory cytokine release (e.g., IL-6, TNF-α) [8]. Subclinical hypothyroidism (TSH 8.12 μIU/mL) may also have impaired cardiac output and metabolic reserve, though treatment was deferred due to normal free T4 levels. Collectively, the interplay of genetic predisposition, infection-related inflammation, and metabolic dysfunction culminated in an unusually aggressive PAH phenotype requiring urgent and coordinated intervention.

Comorbidities as accelerants of PAH

Immune Dysregulation and Infections

DS-associated T- and B-cell dysfunction, compounded by neutrophil chemotaxis deficits, heightens susceptibility to infections [9]. Neonatal sepsis and recurrent pneumonia in our patient likely exacerbated pulmonary inflammation and hypoxia, hastening PAH progression. Aggressive antibiotic therapy and respiratory support mitigated these effects, emphasizing infection control as a cornerstone of PAH management.

Failure to Thrive

Poor weight gain (<5th percentile) reflects the interplay of increased cardiac workload, feeding difficulties, and recurrent infections in DS [10,11]. Nutritional rehabilitation was pivotal in preparing the infant for eventual surgery, demonstrating the need for early dietetic intervention in such cases.

Subclinical Hypothyroidism

Though asymptomatic, elevated TSH levels may impair metabolic and cardiac resilience over time [12,13]. Close endocrine follow-up was prioritized post-stabilization to address this comorbidity.

Management: decoding the therapeutic strategy

Acute Medical Therapy

Initiation of sildenafil (2 mg twice daily) targeted pulmonary vasodilation, leveraging evidence that PAH in Down syndrome infants can remain partially reversible if addressed early [14]. By Day 5 of treatment, oxygen saturations improved to 93-94% on room air, suggesting a favorable response. Additional therapies included antibiotics for pneumonia, ondansetron for vomiting, and bronchodilators, reflecting a comprehensive, multimodal approach to reduce all exacerbating factors contributing to PAH [8,14].

Surgical Deferral

Despite the severity of PAH, ASD closure was deferred, underscoring the heightened perioperative risks in a small, acutely ill infant with active infections [14,15]. Literature supports timely surgical intervention prior to the development of irreversible pulmonary vascular disease, yet in this case, the surgical team determined that immediate repair was too high-risk given the infant’s weight of 3.5 kg and concurrent pneumonia. A “stabilize now, operate later” strategy was adopted, with expectations that weight gain, infection resolution, and continued pulmonary vasodilator therapy would reduce operative morbidity and improve surgical outcomes [14,16].

Multidisciplinary Collaboration

Close coordination among cardiology, pulmonology, infectious disease, nutrition, and cardiothoracic surgery specialists proved crucial. Cardiologists directed PAH management, pulmonologists focused on respiratory support and infection control, while surgeons evaluated the timing of intervention. This team-based strategy ensured that interrelated comorbidities - immune vulnerability, hypothyroidism, and growth deficits - were addressed concurrently, optimizing the patient’s readiness for definitive ASD closure [10,14].

Insights from the literature and clinical implications

Down syndrome infants with congenital heart defects are at particularly high risk for early-onset PAH. Registry data indicate a median age of PH diagnosis at approximately six months in this population [15]. Notably, PH diagnosed before six months of age has a significantly higher likelihood of resolution following surgical repair compared to diagnoses made later (54% vs. 29% resolution) [15]. Our case thus reinforces the value of vigilant echocardiographic follow-up - monthly if necessary - in high-risk infants, particularly those showing growth failure or recurrent infections [10,15].

Therapeutically, the use of sildenafil aligns with emerging evidence supporting pulmonary vasodilators in Down syndrome-associated PAH [14,16]. Although the optimal dosing regimen remains under investigation, early administration in our patient facilitated clinical stabilization, enabling deferment of surgery until less hazardous circumstances arose. From a surgical standpoint, timing remains a delicate balance: early ASD closure may arrest PAH progression, but operating on a critically ill infant with ongoing infections and malnutrition poses significant perioperative risks [16]. This case illustrates the prudence of a staged strategy - initial medical stabilization followed by definitive curative intervention.

The rapid evolution of severe PAH in this six-month-old with Down syndrome and a secundum ASD provides a stark reminder that Down syndrome can significantly modify the natural course of congenital heart lesions [17]. A convergence of factors - recurrent infections, immune dysregulation, and subclinical hypothyroidism - alongside a substantial left-to-right shunt, created a high-stakes scenario necessitating urgent pharmacologic intervention and meticulously timed surgical planning [8,9,11,18]. By prioritizing infection control, nutritional support, and pulmonary vasodilation, the multidisciplinary team aimed to optimize the infant’s condition for eventual ASD closure. Ultimately, this case underscores the critical importance of early and frequent cardiac evaluation in infants with Down syndrome, aggressive management of associated comorbidities, and adaptable intervention strategies - a model that may improve outcomes in similarly complex pediatric cases.

## Conclusions

This six-month-old infant with Down syndrome developed severe pulmonary arterial hypertension (PAH) secondary to an ostensibly “benign” secundum atrial septal defect (ASD), illustrating how comorbidities in Down syndrome - recurrent infections, immune dysregulation, and failure to thrive - can precipitate aggressive PAH at an unexpectedly early age. Prompt multidisciplinary management, which included pulmonary vasodilators, infection control, and nutritional support, stabilized the patient and set the stage for planned ASD closure to potentially reverse PAH. Given Down syndrome’s 10-fold higher risk of PAH, vigilant echocardiographic screening is crucial, as timely recognition and intervention can prevent irreversible pulmonary vascular damage. This case underscores the importance of aggressively managing infections, tracking growth and thyroid status, and considering relatively early surgical intervention in Down syndrome infants with significant shunts. Long-term follow-up will ultimately clarify optimal timing for both closure of the defect and tapering of pharmacological support, guiding future management strategies in this vulnerable population.
